# Diminished NAD+ levels and activation of retrotransposons promote postovulatory aged oocyte (POAO) death

**DOI:** 10.1038/s41420-024-01876-w

**Published:** 2024-02-28

**Authors:** Ajay K. Singh, Aradhana Mohanty, S. Lava Kumar, Anjali Kumari, Rohit Beniwal, Ajith Kumar Etikuppam, Pravin Birajdar, Athar Mohd, H. B. D. Prasada Rao

**Affiliations:** 1 National Institute of Animal Biotechnology, Hyderabad, Telangana 500032 India; 2 Graduate studies, Regional Centre for Biotechnology, Faridabad, 121 001 India; 3 Present Address: Department of Ophthalmology, University of Rochester, Rochester, NY 14620 USA

**Keywords:** Apoptosis, Infertility

## Abstract

Death is the fate of postovulatory aged or unfertilized oocytes (POAO) in many animals. However, precise molecular mechanisms are yet to be discovered. Here, we demonstrate that increased amounts of reactive oxygen species (ROS), calcium ion (Ca+2) channels, and retrotransposon activity induce apoptosis, which in turn causes POAO death. Notably, suppression of ROS, Ca+2 channels, and retrotransposons delayed POAO death. Further, we found that the histone H4K12 and K16 acetylation increased via downregulation of NAD+ and NAD+ -dependent histone deacetylase SIRT3. Furthermore, adding NMN, sodium pyruvate, or CD38 inhibition delayed the death of postovulatory aged oocytes. Finally, we demonstrate the conservation of retrotransposon-induced DNA damage-dependent POAO death in higher-order vertebrates. Our findings suggest that POAO mortality is caused by cyclic cascade metabolic interactions in which low NAD+ levels increase histone acetylation by inhibiting histone deacetylases, resulting in an increase in retrotransposons, ROS, and Ca+2 channel activity and thus contributing to DNA damage-induced apoptosis.

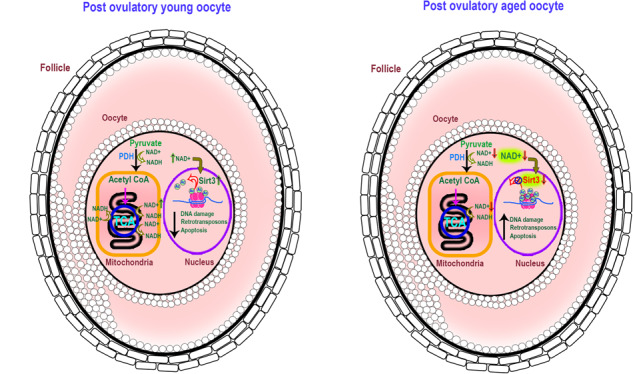

## Introduction

Ovulation is a highly coordinated process that begins with a surge of luteinizing hormone (LH) from the hypothalamic-pituitary-ovarian axis and ends with the disintegration of the mature ovarian follicle to release the fertilizable egg [1]. The released egg then proceeds to the fallopian tube and waits until 12 to 24 h for fertilization [2, 3]. The ovulated egg’s lifespan varies depending on the species. For instance, 12 to 24 h in humans, 8 to 16 h in cattle, and 20 to 26 h in mice [2, 4–8]. Following that, the unfertilized egg undergoes postovulatory ageing, which leads to death [2, 7]. Supporting this, the reports suggest that insemination that occurs on or around the day of ovulation has been linked to the best likelihood of successful pregnancy in humans [9]. Postovulatory oocyte ageing occurs both in vivo and in vitro [7]. Clinical manifestations caused by postovulatory aged oocytes (POAO) in humans and other mammals include low rates of fertilization, poor quality embryos, poor implantation, higher rates of early pregnancy losses, spontaneous abortions, lower rates of live births, and higher rates of offspring abnormalities [6, 7]. For instance, implantation rates were lower in ICSI-generated embryos with older oocytes (>24 h in vitro) than those with younger oocytes [10]. In species like bovine, aged oocytes show an increase in polyspermy, leading to the generation of non-viable embryos [11].

Prior studies have shown that postovulatory aged oocytes display a range of morphological, cellular, and molecular abnormalities, such as zona hardening, shrinkage, blebbing, mitochondrial failure, spindle anomalies, loss of chromosomal integrity or fragmentation, and aberrant lipid peroxidation, increased active oxygen species, and epigenetic changes [2, 7, 12–20]. Oxidative stress, in particular, has been linked to decreased maturation-promoting factors, disturbed calcium homeostasis, mitochondrial dysfunction, spindle abnormalities, and DNA damage in oocytes [2, 3, 16, 21, 22]. Moreover, adding melatonin to the in vitro maturation media improved optimum fertilization in mice-aged oocytes [16, 23–26]. Furthermore, postovulatory oocytes exposed to oxidative and DNA-damaging stimuli exhibited higher levels of caspases and lower levels of anti-apoptotic proteins [2, 7, 16, 27, 28]. The disequilibrium between pro and anti-apoptotic genes, notably Bcl-2 and Bax, in POAO, demonstrates the involvement of the active apoptosis process [7, 20, 28–31].

Cumulus cell-produced paracrine substances, sphingolipids, and ceramides increase mitochondrial dysfunction, possibly leading to POAO mortality [2, 32–34]. This is supported by the finding that inhibiting cumulus cell gap junctions in oocytes increase oocyte survival, implying that cumulus cell secretions play a role in postovulatory oocyte ageing [33, 35]. Moreover, postovulatory oocyte ageing alters Ca2+ pump function and Ca2+ homeostasis in the cytoplasm and mitochondria, regardless of oxidative stress, suggesting that Ca2+ signalling in postovulatory aged oocytes is independent of oxidative stress [2]. Although the physiological and morphological consequences of postovulatory oocyte ageing have been established, the molecular pathways remain unknown. Therefore, this study uses mouse and goat oocytes to investigate the molecular mechanisms behind postovulatory oocyte ageing. We proved that a cyclic cascade of metabolic reactions causes postovulatory oocyte death (model). We also demonstrate that higher-order vertebrates share these mechanisms.

## Results

### Dynamics of postovulatory oocyte death

**Fig. 1:**
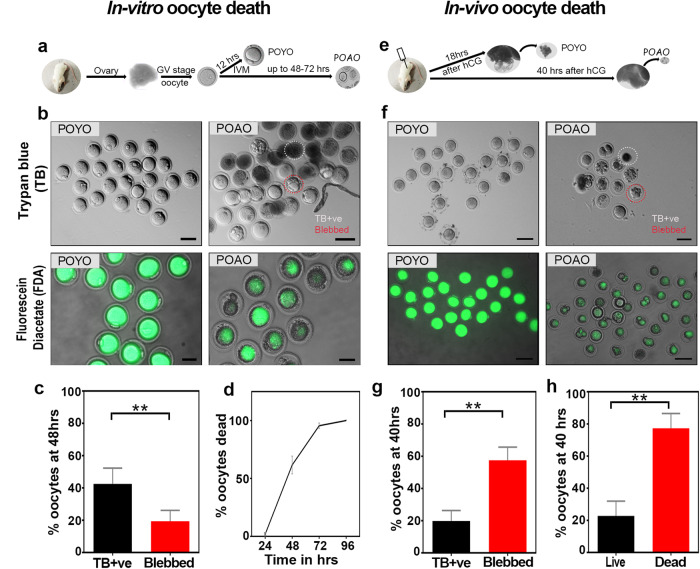
In vitro and In vivo mouse postovulatory oocyte death kinetics. **a**, **e** Schematic representation of postovulatory oocyte ageing. **b** POYO and POAO stained with trypan blue and FDA. **c** Quantification of trypan blue positive (TB +ve) and blebbed oocytes from in vitro maturation (**d**) POAO deathkinetics. **f** In vivo POYO and POAO stained with trypan blue and FDA. **g** Quantification of trypan blue +ve and blebbed oocytes from in vivo maturation. **h** Quantification of % live and dead oocytes from in vivo maturation. More than 500 matured oocytes used to quantify the in vitro death. 150 to 200 oocytes used to quantify the in vivo death. Error bars show mean ± s.e.m **P ≤ 0.008, unpaired *t*-tests. Scale bars = 100 μm.

To better understand the dynamics of the postovulatory aged oocyte death, we cultured GV-stage mouse oocytes, matured them to MII called postovulatory young oocytes (POYO), and then examined the kinetics with trypan blue staining (Fig. 1a, b). Trypan blue dye binds dead cells while preventing live cells from staining [36]. Notably, at the 48-h, 60% of the oocytes displayed positive staining for trypan blue, and this proportion increased to 70% at the 72-h point. Strikingly, even after 96 h, 30% of the oocytes remained unstained by trypan blue (Fig. 1b, d). Intriguingly, within this unstained subset, we observed substantial cytoplasmic blebs in 30% of the oocytes (Fig. 1c). These bleb-containing oocytes exhibited morphological distinctions from others, characterized by unusual shapes (Fig. 1b; Fig. S1a). To achieve greater precision in our analysis of postovulatory aged oocyte (POAO) death dynamics, we utilized fluorescein diacetate (FDA) [36]. Because of the stable cell membranes, the non-polar FDA is permeabilized inside cells and hydrolyzed by esters, resulting in accumulated green fluorescence in live cells. In contrast, dead cells lose fluorescence due to damaged, leaky cell membranes [37]. Almost all POYO are FDA-positive after 24 h (Fig. 1b). At 48 h, the proportion of FDA-positive oocytes dropped to 30%. Further, at 72 h, 100% of the oocytes are FDA-negative, demonstrating that the POAO death occur in a time-dependent manner (Fig. 1b). Both morphologically normal and morphologically abnormal oocytes die within 72 h (Fig. 1d). In order to assess the variations between in vitro and in vivo matured postovulatory aged oocytes death, we also recovered oocytes from the ampulla at 40 h following superovulation (Fig. 1e). In vivo, matured oocytes, like in vitro matured oocytes, have two morphological groups: with and without cytoplasmic blebs (Fig. 1f, g). However, in contrast to in vitro matured oocytes, approximately 80% of in vivo matured oocytes undergo death within 40 h, suggesting a more rapid postovulatory aged oocyte death in the in vivo settings (Fig. 1h).

### Apoptosis-dependent POAO death

**Fig. 2:**
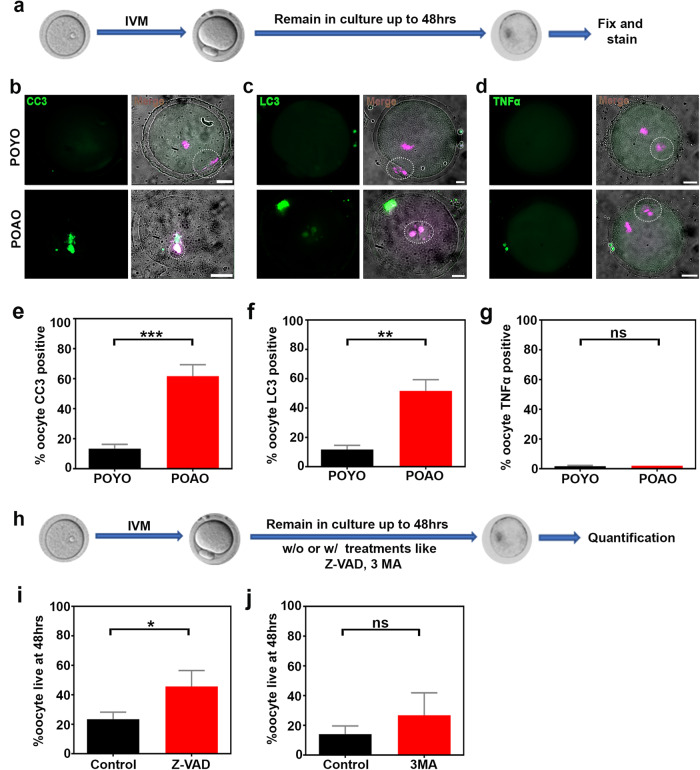
Pathways responsible for POAO death. **a** Schematic representation of postovulatory oocyte ageing. **b** Oocyte nuclei of respective stages immunostained for DNA marker DAPI (magenta) and CC3 (green). **c** Oocyte nuclei of respective stages stained for DNA marker DAPI (magenta) and LC3 (green) (**d**) Oocyte nuclei of respective stages stained for DNA marker DAPI (magenta) and TNFα (green). **e**, **f**, **g** Quantification of CC3, LC3 and TNFα in POYO and POAO. **h** Schematic representation of postovulatory oocyte ageing and treatment. **i**, **j** Quantification of % live POAO at 48 h in the presence and absence of ZVAD, and 3MA. 20 to 25 oocytes were analyzed for localizations. 130 to 150 oocytes were analyzed for each treatment. Error bars show mean ± s.e.m ***P ≤ 0.0005, **P ≤ 0.001, *P ≤ 0.03, (n.s.) *P* ≥ 0.01, unpaired *t*-tests. Scale bars = 10 μm.

Further, we sought to understand the death pathways implicated in the postovulatory aged oocytes death. To identify the POAO death pathways, we immunostained the POYO and POAO with the apoptosis marker cleaved caspase3, the autophagy marker LC3, and the TNF- marker for necrosis (Fig. 2a–d). 55% of the POAO have cleaved caspase3 signals, whereas 45% of POAO show LC3 staining (Fig. 2e, f). We could not detect any TNF-α signs in POAO, indicating that apoptosis is the primary pathway responsible for postovulatory aged oocytes death, whereas autophagy plays a secondary role (Fig. 2e–g). To confirm the functions of apoptosis and autophagy, we introduced the caspase3 inhibitor zVAD-fmk and the autophagy inhibitor 3 MA into POYO after maturation [38, 39] (Fig. 2h). As previously reported, zVAD-fmk binds and inhibits several caspases to prevent apoptosis [40]. In contrast, 3 MA prevents autophagy via inhibiting PI3K [41]. Intriguingly, the POAO death was delayed by adding zVAD-fmk compared to the control (Fig. 2i). At the same time, 3 MA has a negligible impact on POAO life indicating that apoptosis may be a primary death pathway of POAO (Fig. 2j). In addition, oocytes treated with zVAD and 3-MA exhibit slightly elevated levels of class I spindles compared to control POAO, suggesting that delaying postovulatory oocyte ageing by blocking the death pathways may somewhat influence spindle quality (Fig. S4a, b).

### Increased DNA damage in POAO

**Fig. 3:**
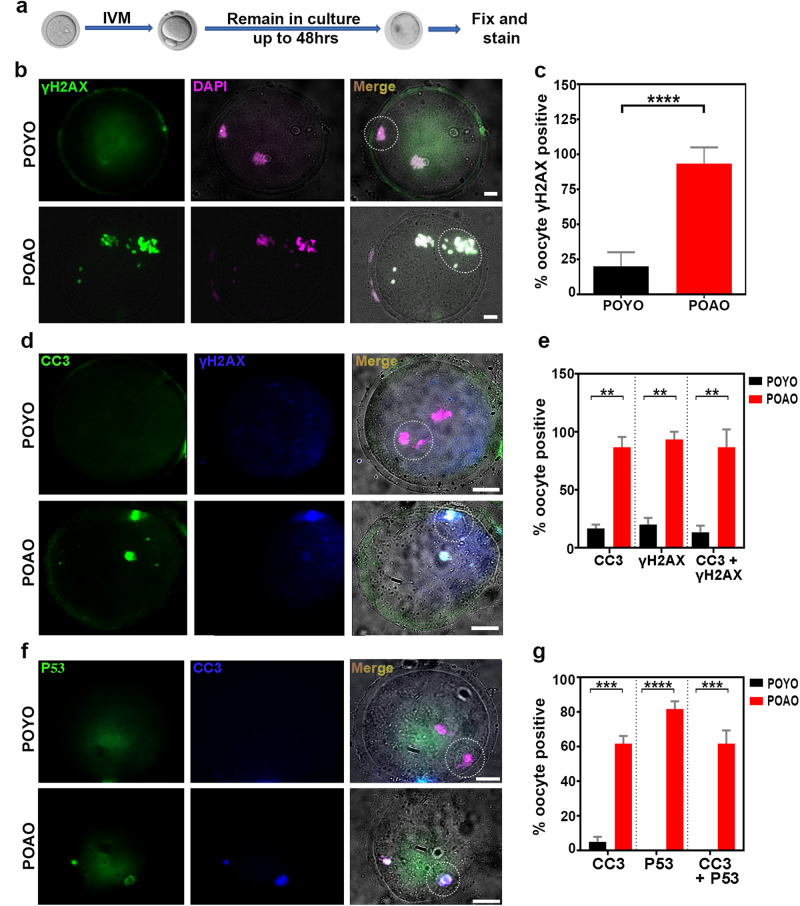
Hyper DNA damage in POAO. **a** Schematic representation of postovulatory oocyte ageing. **b** POYO and POAO nuclei were immunostained for DNA damage marker γH2AX (green) and DNA (magenta). **c** Quantification of γH2AX positive nuclei in respective ages. **d** POYO and POAO nuclei were immunostained for CC3 (green), DNA damage marker γH2AX (blue) and DNA (magenta). **e** Quantification of γH2AX and CC3 colocalized nuclei in respective ages. **f** POYO and POAO nuclei were immunostained for CC3 (blue), p53 (green) and DNA (magenta). **g** Quantification of p53 and CC3 colocalized nuclei in respective ages. 20 to 25 oocytes were analyzed for localizations and colocalizations. Error bars show mean ± s.e.m ****P ≤ 0.00001, ***P ≤ 0.0004, **P ≤ 0.001. A dotted circle indicates the polar body. Scale bars = 10 μm.

We investigated the potential elevation in DNA damage in post-ovulatory aged oocytes (POAO), given that apoptosis stands out as a predominant mechanism of cell death in POAO, with 30% of oocytes displaying morphological abnormalities such as blebbing. To assess the extent of DNA damage in POAO, we employed immunostaining with the DNA damage marker γH2AX on both POYO and POAO, as established by previous studies [42, 43] (Fig. 3a, b). Notably, the nucleus and polar body of 80% of POAO exhibited signs of damaged DNA (Fig. 3b, c). Moreover, our findings revealed an increase in DNA damage in both blebbed and non-blebbed POAO (Fig. 3c). Additionally, we conducted colocalization studies of cleaved caspase3 and γH2AX in POAO to elucidate the association between apoptosis and DNA damage (Fig. 3d). Approximately 70% of POAO displayed colocalization of cleaved caspase3 and γH2AX, suggesting that POAO death is triggered by DNA damage (Fig. 3e). Given the well-established role of the P53 checkpoint protein in inducing cell cycle arrest and activating apoptosis in fully grown oocytes [44]. Thus, we immunostained POYO and POAO with P53 to understand the role of P53 in POAO death (Fig. 3f). Compared to POYO, 60% of POAO exhibited P53 localization, and most P53-positive POAO also displayed γH2AX localization, providing evidence for P53 checkpoint-dependent death in POAO (Fig. 3g).

### Calcium channel and retrotransposon inhibition delayed the postovulatory aged oocytes death

**Fig. 4:**
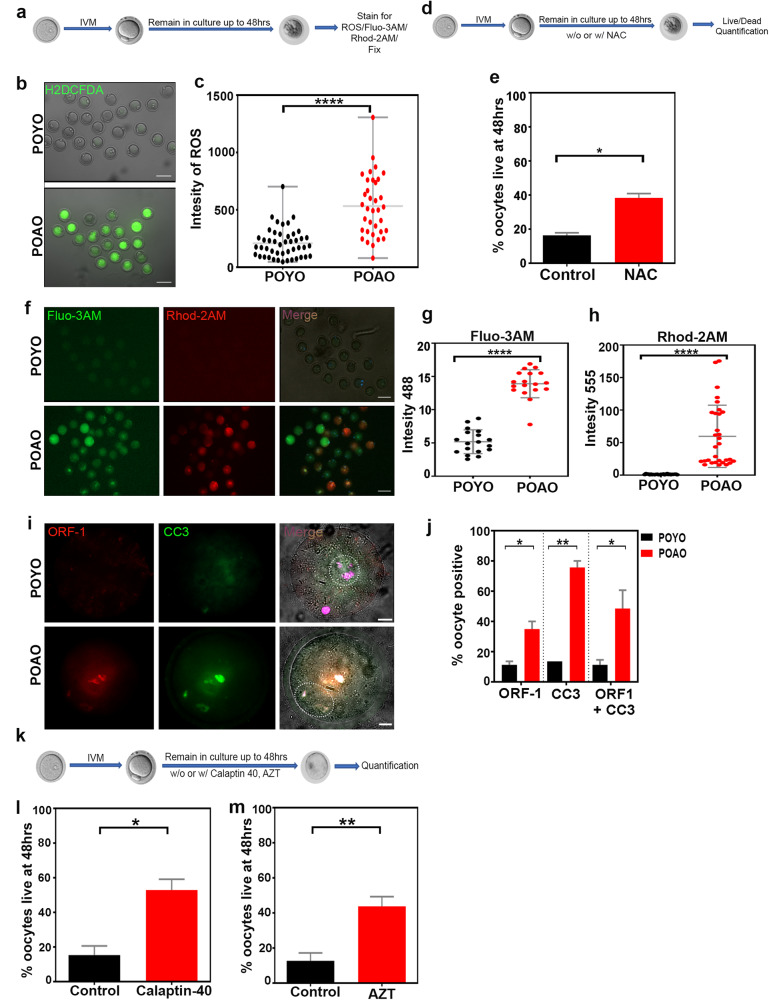
Retrotransposons and calcium levels upregulated in POAO. **a** Schematic representation of postovulatory oocyte ageing and staining. **b** POYO and POAO stained for ROS (green). **c** Quantification of signal intensities of ROS. **d** Schematic representation of postovulatory oocyte ageing and treatments. **e** Quantification of % live POAO at 48 h in the presence and absence of NAC. **f** POYO and POAO stained for Fluo-3 AM (green) and Rhod-2 AM (red). **g**, **h** Quantification of Fluo-3AM and Rhod-2 AM intensities in POAO and young MII oocytes. **i** POYO and POAO stained for CC3 (green) and ORF1 (red). **j** Quantification of ORF1, CC3 alone and colocalization in POYO and POAO. **k** Schematic representation of postovulatory oocyte ageing and treatments. **l**, **m** Quantification of % live POAO at 48 h in the presence and absence of AZT and calpatin-40. More than 200 oocytes used for ROS and Ca+2 analysis. 20-25 oocytes used for ORF1 localization. 80 to 100 oocytes used for treatments. Error bars show mean ± s.e.m ****P ≤ 0.00001, **P ≤ 0.002, *P ≤ 0.04, unpaired *t*-tests. Scale bars = 10 μm.

According to earlier observations, the POAO exhibits enhanced Ca+2 signalling and ROS activity [2, 23, 45]. We, therefore, examined the ROS in POAO to see if the elevated ROS involved in POAO death (Fig. 4a, b). After 48 h, we discovered that POAO had higher ROS activity than POYO (Fig. 4c). To determine the effect of increasing ROS on postovulatory aged oocytes death, we cultured the POYO with or without the ROS scavenger N-acetyl-l-cysteine (NAC). Addition of NAC partially increased the POAO life, indicating that ROS may contribute to POAO death (Fig. 4d). Furthermore, to understand the role of Ca+2 in POAO, we stained POYO and POAO with Ca+2 markers Fluo-3 AM and Rhod-2 AM [46] (Fig. 4a, f). As previously observed, Fluo-3 AM has higher fluorescence binding with cytoplasmic Ca+2, whereas Rhod-2 AM exhibits greater fluorescence binding with mitochondrial Ca+2 [47]. Intriguingly, Ca+2 signals increased in POAO cytoplasm and mitochondria compared to POYO (Fig. 4f–h). Verapamil, a Ca+2 channel blocker, also delayed postovulatory aged oocytes death, demonstrating that Ca+2 signalling is implicated in POAO DNA damage and death (Fig. 4k, l) [48]. Moreover, ageing-related epigenetic changes result in retrotransposon derepression and DNA damage [49–51]. Specifically, an increase in L1 and IPA expression was seen in physiologically aged mice oocytes, highlighting the likelihood of involvement of retrotransposons in POAO death [52]. We therefore immunostained POYO and POAO with ORF1 to explore this possibility (Fig. 4i). As previously reported, ORF1 and ORF2 polypeptides encode for LINE-1 retrotransposon in mammals [53]. Notably, 55% of POAO had ORF1 localization against 10% of POYO (Fig. 4j). Furthermore, the addition of AZT, a known LINE-1 inhibitor, slowed the postovulatory aged oocytes death, demonstrating that POAO death may be triggered by retrotransposon-dependent POAO DNA damage (Fig. 4k, m).

### Histones hyperacetylated during postovulatory oocyte ageing

**Fig. 5:**
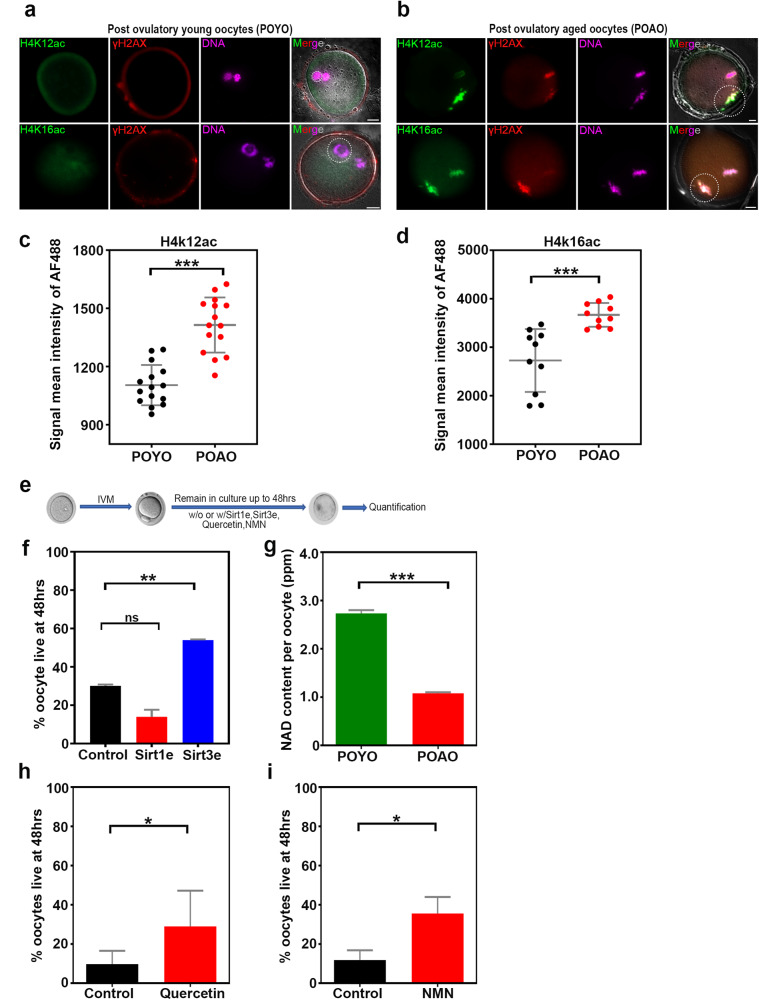
Epigenetic modifications and NAD metabolism regulates the POAO death. **a**, **b** POYO and POAO stained for histone modifications H4K12 or 16 ac (green), DNA damage marker γH2AX (red) and DNA (magenta). **c**, **d** Quantification of H4K12, 16ac signal intensities in POYO and POAO. **e** Schematic representation of postovulatory oocyte ageing and treatments (**f**) Quantification of % live POAO in the presence and absence of SIRT1 and SIRT3 enhancers. **g** Quantification of NAD+ in POYO and POAO. **h**, **i** Quantification of % live POAO in the presence and absence of CD38 inhibitor quercetin and NMN. 20–25 oocytes used for H4K12 and 16ac localization. 80 to 100 oocytes used for treatments. Error bars show mean ± s.e.m ***P ≤ 0.0001, **P ≤ 0.004, *P ≤ 0.03, (n.s.) *P* ≥ 0.01, paired *t*-tests. Scale bars = 10 μm.

Epigenetic alterations have been linked to postovulatory oocyte ageing [18, 54]. Specifically, changes in the methylation of specific genes are time-dependent [18, 55]. The overall increase in histone acetylation in POAO was detected regardless of in vitro or in vivo origin [54]. Additionally, transcriptional activation of the transposable elements is driven by histone acetylation, particularly H4K16ac [56]. For this reason, we thoroughly investigated the histone methylation and acetylation patterns in POYO and POAO (Figure S2a). The histone methylation pattern showed modest variations, as previously published [18] (Figure S2a). The histone acetylation marks, however, were increased in POAO. Notably, the H4K12ac and H4K16ac marks were elevated and colocalized with the DNA damage marker γH2AX, suggesting that hyperacetylation plays a role in postovulatory oocyte ageing (Fig. 5a–d).

### NAD + -dependent histone deacetylase SIRT3 responsible for hyperacetylation

We hypothesized that increased histone acetylation during postovulatory oocyte ageing might result from enhanced histone acetylation or decreased histone deacetylation activities. We specifically targeted the NAD+ -dependent histone deacetylases SIRT1 and SIRT3 to determine the influence of histone deacetylation during postovulatory oocyte ageing [57] (Fig. 5e). The addition of ε viniferin, a SIRT3 enhancer, to the POYO, slowed oocyte ageing [58] (Fig. 5f). In contrast, POAO death dynamics were unaffected by the addition of the SIRT1 enhancer to POYO, suggesting that SIRT3-dependent histone deacetylation may be critical for POAO (Fig. 5f).

### NMN and CD38 inhibitor supplementation delayed the POAO death

SIRT3 is an NAD+ -dependent histone deacetylase [57]. The decrease in SIRT3 in POAO could be attributed to decreased NAD+ levels. In addition, a previous studies have shown that NMN increases quality and competence in postovulatory aged oocytes and maternally aged mice oocytes by replenishing NAD+ levels. As a result, we evaluated NAD+ levels in POYO and POAO to determine the effect of NAD+ on postovulatory aged oocytes death. Contrary to POYO, POAO exhibits a two-fold decrease in NAD+ levels (Fig. 5g). Additionally, the POAO NAD+/NADH ratio rose, suggesting an increased production of ROS and the potential function for NAD+ in postovulatory oocyte ageing. In addition, adding NMN or NADase CD38 inhibitor to the culture media partially delayed the postovulatory aged oocytes death, implying that depleted NAD+ levels may induce POAO death (Fig. 5h, i).

### Sodium pyruvate supplementation enhances the POAO life

**Fig. 6:**
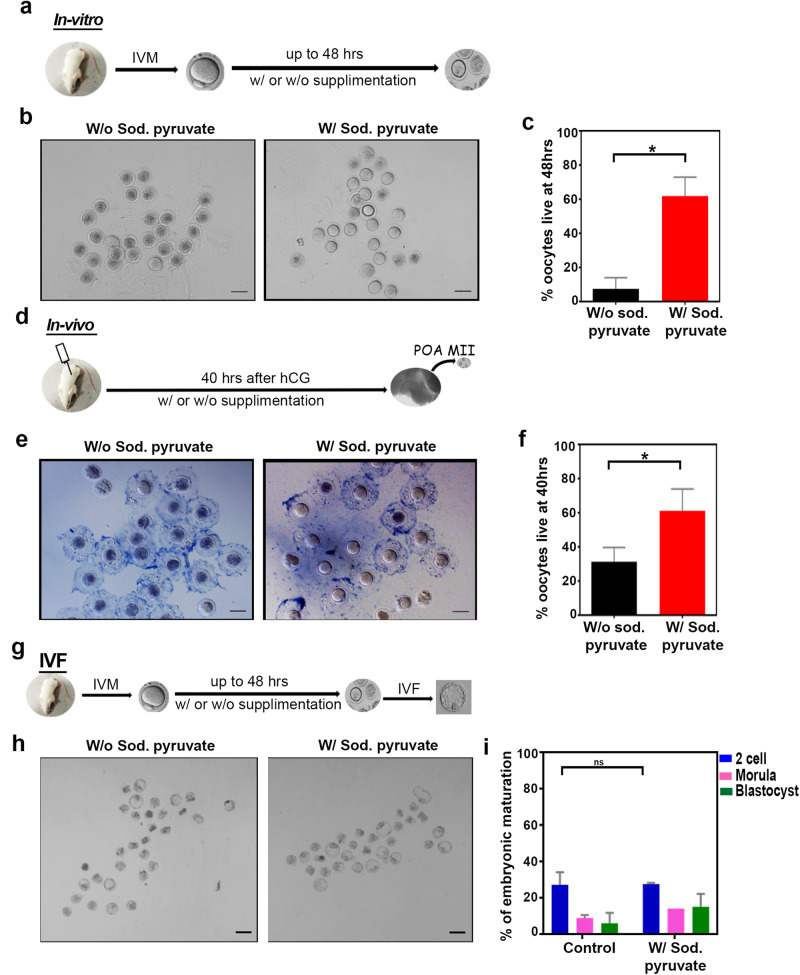
Sodium pyruvate enhances the POAO life without compromising the quality. **a** Schematic representation of oocyte treatments in vitro. **b** Trypan blue staining of POAO with or without sodium pyruvate. **c** Quantification of % live POAO in the presence and absence of sodium pyruvate at 48 h. **d** Schematic representation of oocyte treatments in vivo. **e** Trypan blue staining of POAO with or without sodium pyruvate. **f** Quantification of % live POAO with or without sodium pyruvate oral gavage at 40 h. **g** Schematic representation of IVF. **h** Embryo maturation efficiencies of oocytes POAO with or without sodium pyruvate. **i** % embryo maturation. Error bars show mean ± s.e.m, *P ≤ 0.02, paired *t*-tests. More than 200 oocytes were quantified for each treatment. Scale bars = 100 μm.

Age-related imbalance in NAD+ metabolism contributes to increased breakdown of NAD+. Exogenous pyruvate supplementation has been identified as a potential intervention to counteract ageing by preserving NAD+ metabolism [59–61]. In light of these findings, we sought to investigate whether the addition of pyruvate could mitigate the death of postovulatory aged oocytes, and thus, sodium pyruvate was introduced into the POYO (Fig. 6a). Remarkably, introducing sodium pyruvate resulted in a four-fold increase in the survivability of postovulatory aged oocytes (Fig. 6b, c). To assess the impact of dietary sodium pyruvate on the postovulatory aging of matured oocytes in vivo, we administered sodium pyruvate to mice following superovulation via oral gavage (Fig. 6d). Oocytes were retrieved from the ampulla at 40 h, and the quantification revealed a two-fold increase in live oocytes in the sodium pyruvate-treated ampulla compared to the control (Fig. 6e, f). These findings indicate that sodium pyruvate extends the life of POAO both in vitro and in vivo. To delve deeper into the implications of extended postovulatory oocyte life, we conducted in vitro fertilization (IVF) on sodium pyruvate-rescued oocytes, followed by a comprehensive analysis of subsequent embryo development (Fig. 6g). 70% of oocytes, rescued through sodium pyruvate intervention, successfully progressed to the blastocyst stage, indicating that the quality of the oocyte may not be compromised by delayed postovulatory oocyte ageing (Fig. 6h, i).

### Evolutionarily conserved POAO death

**Fig. 7:**
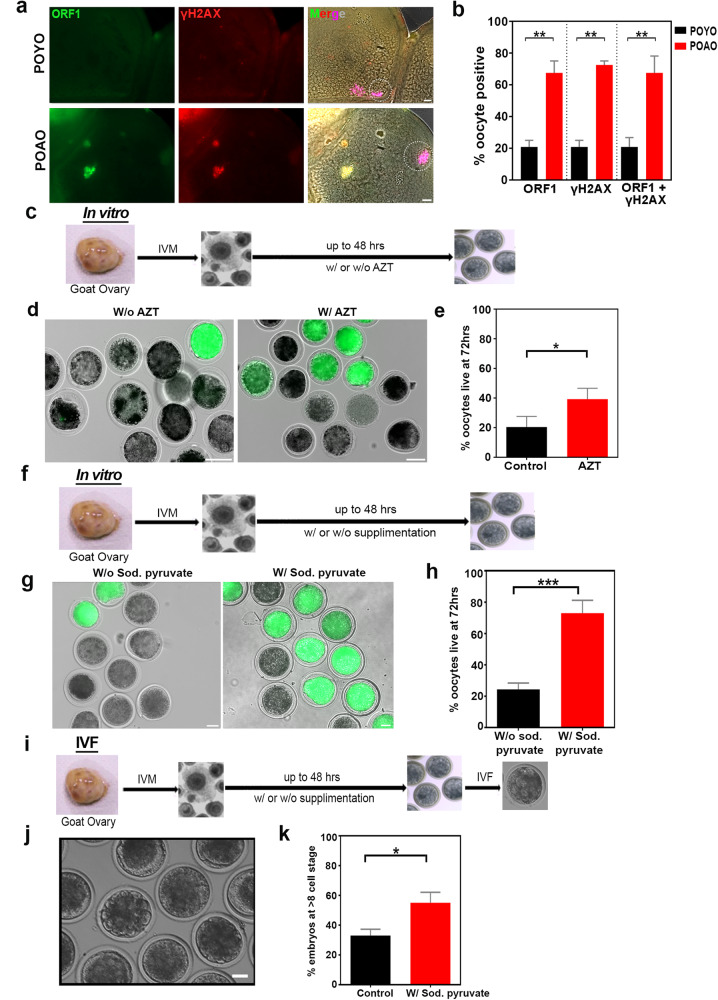
Evolutionary conservation of sodium pyruvate-dependent delayed POAO death. **a** POYO and POAO nuclei were immunostained for ORF1 (green), γH2AX (red) and DNA (magenta). **b** Quantification of ORF1 and γH2AX colocalized nuclei in respective ages. **c** Schematic representation of goat oocyte IVM followed by POAO with or without AZT. **d** POAO with or without AZT stained for FDA (green). **e** Quantification of % live POAO with or without AZT at 72 h. **f** Schematic representation of goat oocyte IVM followed by POAO with or without sodium pyruvate. **g** POAO with or without sodium pyruvate stained for FDA (green). **h** Quantification of % live POAO with or without sodium pyruvate at 72 h. **i** Schematic representation of goat oocyte IVM followed by POAO with or without sodium pyruvate and IVF, embryo maturation. **j** Bright field images of goat embryo (**k**) Quantification of embryo development of goat POAO with or without sodium pyruvate. 20 to 25 oocytes were analyzed for localizations and colocalizations. Error bars show mean ± s.e.m ***P ≤ 0.0001, **P ≤ 0.004, *P ≤ 0.03, unpaired *t*-tests. Scale bars = 100 μm.

To evaluate the evolutionary conservation of POAO death processes in higher-order vertebrates, we examined the POAO death kinetics of in vitro matured, grade I goat oocytes with trypan blue and FDA staining (Fig. S3a–c). POAO death dynamics in goat-matured oocytes are comparable to those in mouse-matured oocytes (Fig. S3d). As mentioned earlier, increased DNA damage is responsible for the death of mouse postovulatory aged oocytes (POAO). We wanted to find out if similar processes contribute to the death of goat POAO. To evaluate DNA damage in goat POAO, we used immunostaining to mark DNA damage indicator γH2AX in both POYO and POAO goat oocytes (Fig. 7a). Similar to what we observed in mice, the analysis revealed a three-fold increase in the localization of γH2AX on nuclear and polar body DNA in goat POAO, suggesting that DNA damage is elevated in goat POAO oocytes (Fig. 7a, b). Additionally, we co-localized γH2AX with ORF1 to investigate the involvement of retrotransposons (Fig. 7a). Just like γH2AX, ORF1 exhibited a three-fold increase in localization in goat POAO (Fig. 7a, b). Notably, a significant portion of γH2AX signals co-localized with ORF1 (Fig. 7a, b). To explore the impact of retrotransposons on POAO death, we introduced AZT, a known LINE-1 inhibitor (Fig. 7c, d). Adding AZT slowed down the death of postovulatory aged goat oocytes, suggesting a potential role of retrotransposons in POAO DNA damage and death (Fig. 7d, e). Furthermore, we conducted experiments with fresh goat post-matured oocytes, both with and without sodium pyruvate (Fig. 7f, g). Like mouse POAO treated with sodium pyruvate, goat POAO with sodium pyruvate exhibited delayed death (Fig. 7g, h). When these life-extended oocytes were subjected to in vitro fertilization (IVF), 55% of those rescued from POAO death by sodium pyruvate developed into 8-cell embryos or later stages, indicating that higher-order vertebrates have conserved mechanisms that may contribute to the death of POAO (Fig. 7i–k).

## Discussion

### Responsible death pathways for postovulatory aged oocyte death

Understanding the root cause of POAO death holds significant importance, given its occurrence in both in vivo and in vitro scenarios, with potential implications for fertility impairment. Our investigation reveals a notable disparity in the survival rates of in vivo matured POAO compared to their in vitro counterparts. Moreover, we noticed seasonal variations in POAO death (data not shown). In the controlled environment of in vitro maturation, characterized by consistent media composition and atmospheric conditions, the death of POAO unfolds in a time-dependent fashion. This raises a crucial inquiry: What precisely governs the mechanism leading to the death of POAO? Previous studies have pinpointed apoptotic markers, including heightened dense vesicles, ooplasmic alterations, and DNA fragmentation, in both in vivo and in vitro matured oocytes [2]. However, the precise mechanism of the POAO death is unknown. Our study proposes that POAO activates cell death pathways primarily relying on apoptosis. Specifically, the introduction of zVAD-fmk demonstrates a more pronounced delay in POAO death than 3-MA, suggesting that apoptosis takes precedence as the predominant mechanism, followed by autophagy. This conclusion is supported by the observed localization patterns of cleaved caspase 3 and LC3, further substantiating the central role of apoptosis in the process. Consistent with our findings, previous studies have indicated a decrease in the anti-apoptotic factor BCL2 in mice and pig POAO, suggesting that apoptosis may be the primary pathway implicated in POAO death [29].

### DNA damage-dependent postovulatory aged oocyte death

Given that apoptosis is the most common form of POAO death, we sought to investigate what causes apoptosis. Apoptosis is a type of programmed cell death that occurs due to increased cellular stress and DNA damage throughout development and ageing [62]. At the cellular level, damaged DNA that is not repaired rapidly may result in cell death [63]. Therefore, we examined the DNA damage in POYO and POAO and found that DNA damage is increased in POAO. Further, we found that the DNA damage marker γH2AX colocalizes with cleaved caspase3, indicating that increased DNA damage might be the reason for apoptosis. Since the cellular stress might be a precursor to DNA damage, we investigated ROS levels. Our findings indicate a surge in ROS activity in POAO, implying a potential contribution of ROS to DNA damage. Introducing N-acetylcysteine (NAC) partially delayed programmed cell death in mice and goat POAO, suggesting that additional pathways may be implicated in DNA damage or the repair mechanisms might be inactive in POAO. As previously noted, our investigation identified an augmented cytoplasmic calcium flow in POAO [64]. Under specific circumstances, excessive calcium levels can lead to mitochondrial membrane permeability, resulting in the release of apoptotic agents [64]. Furthermore, cells may initiate apoptosis in response to DNA damage by triggering ER-mitochondria Ca2+ transfer [65]. On the other hand, in biologically older animals, the diminishment of heterochromatin histone marks adversely impacts oocyte quality by upregulating the expression of L1 and IAP retrotransposons, leading to DNA damage [52]. Recent studies also indicate that transposable elements may undergo derepression due to age-related epigenetic alterations [66]. Moreover, LINE-1 unsilencing in cells induces apoptosis or a condition resembling cellular senescence through increased DNA damage, suggesting that retrotransposons may cause the excessive DNA damage seen in POAO [67]. In POAO, as opposed to POYO, we observed an elevation in ORF1 localization. Additionally, in POAO, ORF1 colocalizes with cleaved caspase-3. Introducing the retrotransposon inhibitor AZT to the medium delayed ageing of post-ovulatory oocytes, indicating a potential function for retrotransposons in maintaining genome integrity in post-ovulatory aged oocytes.

### Role of histone modifications in postovulatory oocyte ageing and its impact on energy metabolism

As outlined above, histone modifications play a crucial role in the derepression of retrotransposons during post ovulatory oocyte ageing [68]. This prompts the question: what changes occur in histone modification during POAO? Earlier studies indicate postovulatory oocyte ageing is associated with increased histone acetylation, specifically at H3K14, H3K8, and H4K12 [30]. Furthermore, the addition of TSA enhanced the oocyte ageing by elevating histone acetylation [30]. In contrast, caffeine treatment reduces acetylation, thereby delaying oocyte ageing [54]. Our investigation into H3 and H4 acetylation and methylation marks in both POYO and POAO revealed an elevation in H4K12 and H4K16 acetylation during POAO. Additionally, hyperacetylated H4K12 and H4K16 were found to colocalize with the DNA damage marker γH2AX. Previous findings suggest that increased H4K12 and K16 promote retrotransposon transcription, indicating that histone hyperacetylation may contribute to elevated retrotransposon activity, leading to DNA damage [56]. The mechanism behind age-related histone acetylation increase involves two potential outcomes: an increase in histone acetyltransferase activity or a decrease in deacetylase activity. Past research indicates that enhancing SIRT1 activity can partially alleviate oocyte maturation abnormalities in physiologically aged animals [69]. Similarly, our observations suggest that adding a SIRT3 enhancer delayed oocyte death but not the SIRT1 enhancer, implying that SIRT3-dependent hyperacetylation may be involved during POAO ageing. Since SIRT3 is an NAD + -dependent histone deacetylase, we measured NAD+ levels in both POYO and POAO and found a two-fold lower NAD+ level in POAO. Furthermore, the partial postponement of POAO death by adding NMN or inhibiting NADase suggests that POAO hyperacetylation may be caused by NAD+ -dependent SIRT3 activity.

NAD+ influences several processes, including energy metabolism, epigenetic changes, DNA repair, ageing, and cell death [59, 60]. For example, NAD+ is a crucial redox carrier that accepts hydride from metabolic processes such as glycolysis, the TCA cycle, and fatty acid metabolism to generate NADH [70, 71]. NADH, in turn, acts as a major hydride donor for ATP synthesis through mitochondrial OXPHOS [70]. Additionally, NAD+ is also a cofactor for several hydrolases, PARPs, and HDACs emphasizing its significance in maintaining cellular equilibrium for development and survival [60]. For instance, persistent PARP activation during DNA damage may result in NAD+ depletion and cell death [60]. CD38 utilizes NAD+ to activate calcium-releasing second messengers, contributing to age-related NAD+ decline, a phenomenon well-established in the aging process [72]. Similarly, our findings reveal lower NAD+ levels in POAO than in POYO, suggesting that POAO shares comparable NAD + -dependent ageing mechanisms with somatic cells. NAD+ is produced in mammalian cells via many processes. (1) Tryptophan, a dietary amino acid, is used in the de novo synthesis route to produce NAD+. (2) The Salvage route recycles NAD+ from NAM, NA, NR, and NMN to keep cellular NAD+ levels stable. (3) Endogenous oxidation of NADH by pyruvate reduction produces NAD+ [73]. As a result, pyruvate is being considered for anti-ageing therapy [61]. Previous research indicates that pyruvate supplementation slows the ageing of in vitro matured mouse oocytes [74]. Our research further supports this, showing that oral gavage of pyruvate delays oocyte death in vivo, suggesting its potential to slow postovulatory oocyte ageing. Finally, we establish the conservation of POAO death mechanisms in higher vertebrates, underscoring the broader relevance of our findings.

## Materials and methods

### Animal Ethics

The mice used in this study were FVB strain 8 to 10 weeks old and weighed about 20–25 g. According to the Institutional Animal Ethics Committee guidelines, the mice were housed under a 12 h light/12 h dark cycle at a constant temperature of 21–22 °C with food and water supplied ad libitum.

### Mice oocyte collection and In vivo, In vitro ageing

Following euthanasia, ovaries were surgically extracted from female mice. Germinal vesicle (GV) stage oocytes were obtained by puncturing visible follicles with a 30-gauge needle in pre-warmed (37 °C) M2 media. In vitro oocyte maturation (IVM) culture was performed in 100 µl droplets of M2 media in a 35 mm culture dish containing 25–30 oocytes per droplet and overlaid with mineral oil. The culture was maintained at 37 °C under 5% CO2 in humidified air for 10–12 h. MII oocytes were then isolated based on polar body extrusion. Subsequently, matured oocytes were aged under the same culture conditions at different time points as experimental requirements.

A series of carefully orchestrated steps were implemented to retrieve in vivo matured oocytes from the ampulla of the fallopian tube. In the evening, female mice were subjected to intraperitoneal administration of 10 IU of PMSG (MSD-Folligon®). Following a 48-hour interval, another intraperitoneal administration of 10 IU of HCG (MSD-Chorulon®) was carried out. The subsequent morning, post-HCG administration was designated as the precise time for harvesting freshly matured oocytes. The hormonally stimulated mice were then allowed to age under consistent conditions to get the aged oocytes.

### Goat oocyte collection, culture, and In vitro ageing

Osmanabadi breed goat ovaries were collected from a nearby local slaughterhouse and transported in PBS quickly to the lab. The oocytes were collected by slicing and puncturing the ovary, and only oocytes with compact three to four layers of cumulus cells and uniform ooplasm were chosen for further experiments. The oocytes were then cultured in IVM media, which contained TCM199, 10 IU/ml follicle-stimulating hormone (FSH), 10 IU/ml luteinizing hormone (LH), 10% follicular fluid, 10% fetal bovine serum (FBS), and 50 µg/ml penicillin-streptomycin, for 30 h at 38.5 °C under 5% CO_2_ in humidified air. The MII oocytes were isolated based on the COC expansion and Hoechest staining. According to experimental requirements, the culture was then extended further at different time points to age them with the same culture condition.

### In vitro fertilization of mouse oocytes

The cauda epididymis of a euthanized male mouse was dissected, and the cauda epididymis was sliced to allow the spermatozoa to emerge in KSOM medium (Sigma- MR-121) supplemented with 10 mg/ml BSA. After 10 min, the supernatant containing highly motile spermatozoa was collected and capacitated for 1 h at 37 °C in the same medium. A concentration of about 1 × 10^6^ sperm cells/ml was prepared. The prepared sperm cells were then used for conventional In vitro fertilization of In vitro/In vivo matured oocytes.

### In vitro fertilization of goat oocytes

Fresh sperm was collected from a male buck in our institutional buck. The recovered sperm was washed with modified Tyrode’s medium (TALP) and centrifuged at 500 g for 5 min before discarding the supernatant. The same method was repeated, and the pellet was ultimately covered with TALP medium. The swim-up method was used to select highly motile spermatozoa, and the final concentration was 1 × 10^7^ ml. The standard IVF procedure involved withdrawing 50 µl of media from the 100 µl droplet created during IVM and replacing it with 50 µl of sperm-containing media. It was then incubated overnight in a CO_2_ incubator with 5% CO_2_ at 38 °C in humidified air.

### Live/dead assay

To determine oocyte death, two methods were used: The Trypan blue dye exclusion viability test and the fluorescein diacetate (FDA) fluorescence test.

#### Trypan blue

0.1% trypan blue was prepared in M2 media to determine oocyte death. POYO and POAO were then incubated in M2 containing 0.1% trypan blue for 10 min and examined under the microscope. The Trypan blue exclusion assay is a common method, where dead cells absorb the dye into their membranes and appear blue.

#### FDA staining

First, a stock solution of FDA was prepared by dissolving 5 mg of FDA (F7378-5G, Sigma) in 1 ml of acetone. To create a working dilution, 2 µl of the 5 mg/ml FDA stock solution was added to 1 ml of M2 media. Then, POYO and POAO were incubated with a working FDA solution at room temperature for 2 min in the dark and images were captured immediately with a fluorescence microscope. FDA is taken up by live cells, which convert the non-fluorescent FDA into the green fluorescent metabolite fluorescein.

### Chemical treatments

To study the effect of the metabolic process on in vitro ageing. Matured oocytes were obtained immediately either after in vitro or in vivo maturation and treated with the following compounds, either individually or in combination under a CO_2_ incubator with 5% CO_2_ at 37 °C in humidified air for another 36–72 h:

#### Z-VAD-FMK (MCE- HY-16658B)

To assess the impact of Z-VAD on postovulatory oocyte ageing, three distinct concentrations (2 μM, 50 μM, and 100 μM) were incorporated into the media. Based on preliminary observations, 2 μM emerged as the optimal concentration for further investigation. For the preparation of stock solutions, 1 mM Z-VAD was dissolved in DMSO and stored in aliquots at −20 °C. Prior to usage, the stock solution was then diluted to the desired concentration using the corresponding ageing media. The ageing culture was conducted in a 35 mm culture dish, with each droplet containing 100 µl of M2 media, maintained at 37 °C under 5% CO2 in humidified air.

#### 3-Methyladenine (3-MA) (TCI-M2518)

To assess the impact of autophagy inhibitor 3-Methyladenine (3-MA) on postovulatory oocyte ageing, three distinct concentrations (0.2 mM, 0.5 mM, and 5 mM) were incorporated into the media. Based on preliminary observations, 0.5 mM used as the optimal concentration for further investigation. To prepare stock solutions, 3-MA (10 mM) was dissolved in M2 medium and stored in aliquots at −20 °C. The stock solution was then diluted to the desired concentration (0.5 mM) with corresponding aging media immediately before use. The ageing culture was performed as described above.

#### Verapamil (Calpatin-40) (Sigma-676777)

To assess the impact of impact of the calcium channel blocker Verapamil (Calpatin-40) on postovulatory oocyte ageing, three distinct concentrations (5 µM, 10 µM, and 15 µM) were incorporated into the media. Based on preliminary observations, 10 µM used as the optimal concentration for further investigation.

To prepare stock solutions, Calpatin-40 (1 mM) was dissolved in M2 medium and stored at −20 °C, then diluted to the desired concentration (10 µM) with corresponding aging media immediately before use. The ageing culture was performed as described above.

#### Azidothymidine (AZT) (Cayman478 15492)

To assess the impact of retrotransposon reverse-transcriptase inhibitor azidothymidine (AZT) on postovulatory oocyte ageing, three distinct concentrations (5 µM, 10 µM, and 15 µM) were incorporated into the media. Based on preliminary observations, 10 µM used as the optimal concentration for further investigation.

1 mM stock solution of AZT was prepared in M2 medium and stored at −20 °C. Before use, it was diluted to a concentration of 10 µM with the corresponding aging media. The ageing culture was performed as described above. Goat oocytes were treated with a concentration of 50 µM in corresponding ageing media after 27 h and cultured at 38.5 °C under 5% CO2 in humidified air.

#### N-acetyl-l-cysteine (NAC) (Sigma-A9165)

To assess the impact of the ROS inhibitor N-acetyl-l-cysteine (NAC) on postovulatory oocyte ageing, a 100 mM stock solution of NAC was prepared in M2 medium and kept at −20 °C. Prior to use, it was diluted to a 1 mM concentration with the corresponding ageing media. The ageing culture was performed as described above. Goat oocytes were treated with a concentration of 1 mM in corresponding ageing media after 27 h and cultured at 38.5 °C under 5% CO2 in humidified air.

#### SRT1720 (MCE- HY-10532)

To assess the influence of the SIRT1 enhancer SRT1720 on postovulatory oocyte ageing, a 1 mM stock solution of SRT1720 was prepared in DMSO and stored at −20 °C. Prior to use, it was diluted to a 0.5 µM concentration with the corresponding ageing media. The ageing culture was performed as described above.

#### Viniferin (Sigma- SMB00074)

To assess the influence of the SIRT3 enhancer Viniferin on the postovulatory oocyte ageing, a 1 mM stock solution of SRT1720 was prepared in DMSO and stored at −20 °C. Prior to use, it was diluted to a 1 µM concentration with the corresponding ageing media. The ageing culture was performed as described above.

#### Quercetin (SRL-71923)

To assess the impact of the CD38 NADase inhibitor Quercetin on the postovulatory oocyte ageing, three distinct concentrations (10 µM, 20 µM, and 30 µM) were incorporated into the media. Based on preliminary observations, 20 µM used as the optimal concentration for further investigation. A 1 mM stock solution of Quercetin was prepared in DMSO and stored at −20 °C. Prior to use, it was diluted to a 20 µM concentration with the corresponding aging media. The ageing culture was performed as described above.

#### Nicotinamide mononucleotide (NMN) (SRL-95130)

To evaluate the effect of the Nicotinamide mononucleotide (NMN) postovulatory oocyte ageing, a 10 mM stock solution of NMN was prepared in M2 media and stored at −20 °C. Prior to use, it was diluted to a 100 µM concentration with the corresponding aging media. The ageing culture was performed as described above.

#### Sodium pyruvate (Sigma-P4562)

To assess the impact of the Sodium pyruvate on the postovulatory oocyte ageing, three distinct concentrations (1 mg/ml, 3 mg/ml, and 5 mg/ml) were incorporated into the media. Based on preliminary observations, 3 mg/ml used as the optimal concentration for further investigation. Every time a fresh 3 mg/ml Sodium pyruvate solution was prepared in M2 media prior to use. Moreover, to assess the impact under in vivo conditions, a solution of Sodium pyruvate at a concentration of 3 mg/ml, dissolved in water, was administered directly to mice via oral gavage for three consecutive days. This treatment commenced one day before ovulation. Subsequently, goat oocytes were subjected to a concentration of 3 mg/ml in the respective ageing media after 27 h and cultured at 38.5 °C under 5% CO2 in a humidified air environment.

### Immunocytochemistry

POYO and POAO were fixed and permeabilized in 4% paraformaldehyde containing 0.05% Triton-X 100 for 30 min. The fixed oocytes were then rinsed three times in PBS containing 0.05% Triton-X100 before being blocked with normal goat serum for 1 h at room temperature. After blocking, the oocytes were incubated with primary antibodies specific for P53 (DSHB-S1-1628, 1:150), cleaved caspase 3 (CST-9661, 1:200), γH2AX (Milipore-05-636, 1:500), LC3 (CST-3868S, 1:200), TNFα (dilution 1:100), LINE-1 ORF1p (dilution 1:100), H3me panel I ((Epigenetics-C10001-1, 1:200) H3K9me2, H3K9me3, H3K27me2, H3K27me3), H3ac panel I ((Epigenetics-C10010-1, 1:200) H3K9ac, H3K27ac), H3ac panel II ((Epigenetics-C10011-1, 1:200) H3K36ac, H3K56ac, H3K79ac), H4K12ac (Epigenetics-A68357-050, 1:200), H4K16ac (Epigenetics-A68398-050, 1:200), in full-strength ADB overnight at room temperature in a humid chamber. The next day, the oocytes were washed three times (5 min each) in PBS containing 0.05% Triton-X100 and then blocked with normal goat serum for 30 min before being incubated with relevant Alexa Fluor-conjugated secondary antibodies diluted 1:5000 in ADB for 1 h at room temperature in the dark. The oocytes were then washed three times (5 min each) in PBS containing 0.05% Triton-X100 and mounted and counterstained with Prolong Gold containing DAPI at room temperature.

### ROS measurement in POYO and POAO

To quantify the ROS levels, POYO and POAO were incubated in M2 media containing 10 μM 2′,7′ dichlorodihydrofluorescein diacetate (H2DCFDA; Invitrogen, D399) for 5 min, followed by three washes with PBS. They were examined under the fluorescence microscope (Axio Observer 7 - Carl Zeiss Microscopy GmbH) for Alexa Fluor (AF) 488. The quantification of fluorescence intensity was measured using Zeiss 2.6 blue edition software.

### NAD+ measurement in POYO and POAO

To determine the ratio of NADH/NAD in POYO and POAO, we collected 200 oocytes for each group. We measured the intracellular levels of NADH and NAD using the NAD/NADH quantitation kit (MAK037; Sigma-Aldrich, St. Louis, MO) according to the manufacturer’s instructions.

### Calcium measurement: Fluo-3AM, Rhod-2AM

Both groups (POYO and POAO) oocytes Calcium levels were measured using Rhod‐2AM (R1245MP, Invitrogen) and Flou‐3 AM (F1242, Invitrogen) according to the manufacturer’s instructions. The oocytes were stained with 5 μM Rhod‐2AM and 5 μM Flou‐3 AM for 30 min in maturation medium and thoroughly washed with DPBS, followed by incubation in maturation medium‐free Rhod‐2AM at 37 °C under a 5% CO_2_ atmosphere for 30 min. Cells were subsequently observed under fluorescent microscope.

### Fluorescence imaging

Immunofluorescence-stained oocytes were imaged using Zeiss Axio scope VII microscope with 10×, 40× Plan Apochromat 0.45 NA, or 63/100×Plan Apochromat 1.4 NA objectives and EXFO X-Cite metal halide light source. Images were captured with a Hamamatsu ORCA-ER CCD camera and processed using Zen software. All comparisons were made between datasets obtained from animals that were matched by age. Two observers performed all quantitative analyses; the second observer was blind to which group was being analyzed.

### Statistical analyses

All data sets were analyzed using a two-tailed unpaired Student’s *t*-test followed by a multiple comparisons test in GraphPad Prism 8. All experiments were performed with at least 3 biological replicates (specified for each set of experiments in results). Unless otherwise stated, all data are presented as mean ± standard error of the mean (SEM) or standard deviation (SD). Statistical significance was represented based on the p value.

### Supplementary information

Suplimentary data

## Data Availability

The data that support the findings of this study are available from the corresponding author upon request.

## Material availability

Further information and requests for resources and reagents should be directed to the lead contact H.B.D. Prasada Rao, prasad@niab.org.in.
