# Supplementary material for: Diminished NAD+ levels and activation of retrotransposons promote postovulatory aged oocyte (POAO) death
Source: Cell Death Discov. 2024 Feb 28;10:104. doi: 10.1038/s41420-024-01876-w (PMC10902361; doi:10.1038/s41420-024-01876-w)
Supplement: Supplementary file 1 — Suplimentary data [file 41420_2024_1876_MOESM1_ESM.pdf]

Fig S1: Morphology of POAO

(a) Bright field images of POAO. Scale bars = 10μm.

Fig S2: Localization of H3 post translational modifications in POYO and POAO

(a) POYO and POAO stained for H3 histone modifications (green) and DNA (magenta). Scale bars = 10μm.

Fig S3: Goat IVM and POAO death kinetics

(a) Goat oocytes selection and in vitro maturation. (b) Quantification of IVM. (c) FDA (green) staining of in vitro matured goat oocytes during ageing at 24, 48 and 76hrs. (d) Quantification of goat in vitro matured aged oocyte death. (e) FDA (green) staining of in vitro matured goat oocytes further incubated with or without NAC. (f) Quantification of % live POAO with or without NAC at 48hrs. Error bars show mean ± s.e.m (*) $P \leq 0.01$ unpaired *t*-tests. GV; Germinal vehicle stage. MI; Metaphase I. MII; Metaphase II. Scale bars = 100μm.

Fig S4: Spindle quality in treatments

(a) POAO with or without treatments stained for tubulin (green) and DNA (magenta). (b) Quantification of different classes of spindles. Class I: Tight spindles; Class II: Loose or circular spindles; Class III: No spindle staining. Error bars show mean ± s.e.m. Dotted white big circles are oocyte and the small circles are polar body. Scale bars = 10μm.

Fig S1:

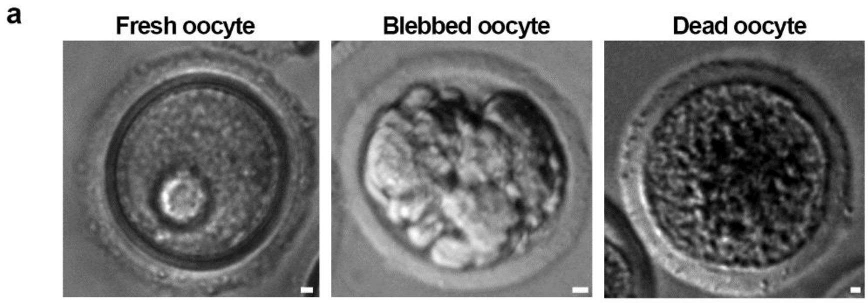

FigS2:

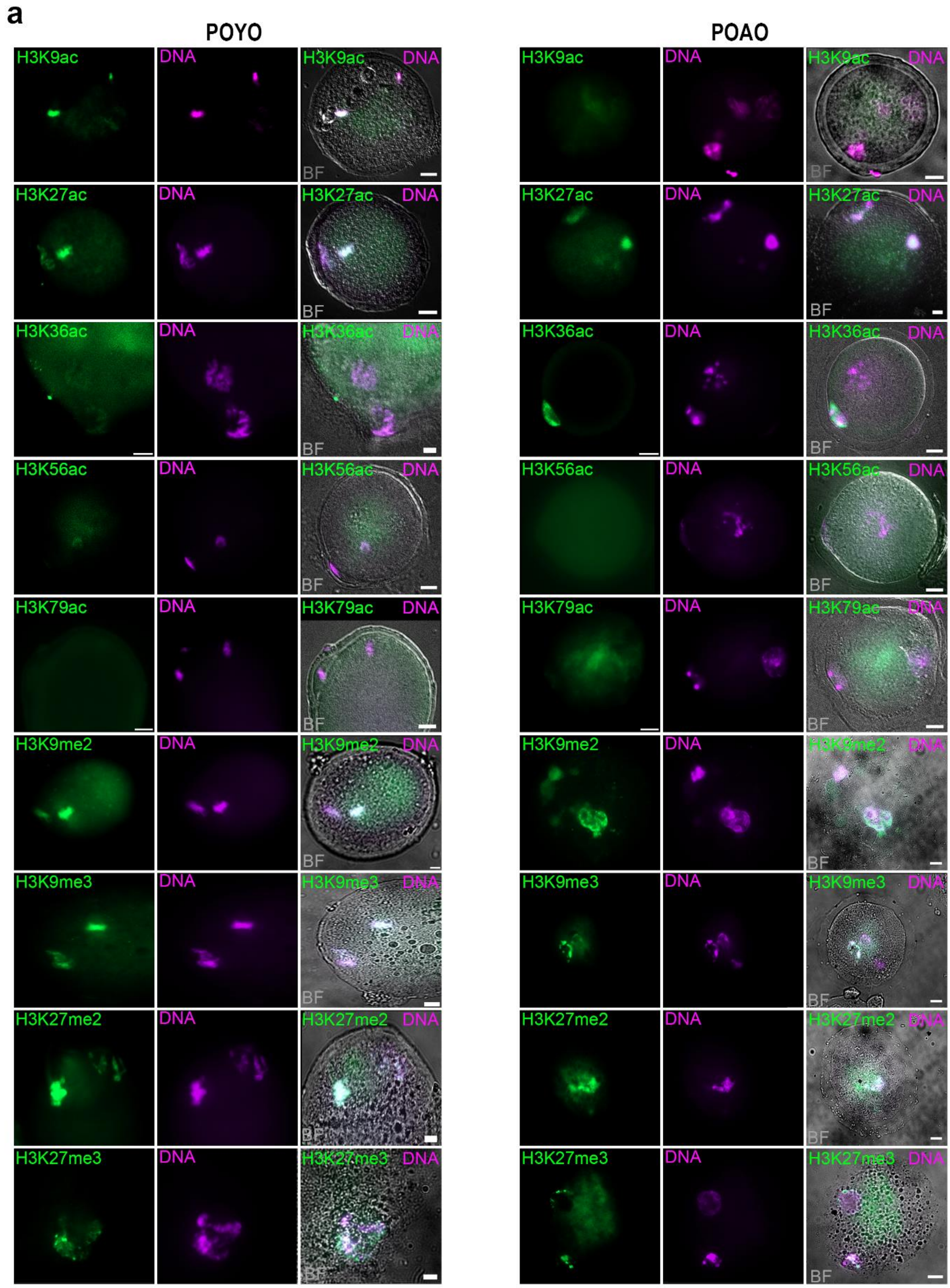
a
POYO
POAO
H3K9ac
DNA
H3K9ac DNA
BF
H3K27ac
DNA
H3K27ac DNA
BF
H3K36ac
DNA
H3K36ac DNA
BF
H3K56ac
DNA
H3K56ac DNA
BF
H3K79ac
DNA
H3K79ac DNA
BF
H3K9me2
DNA
H3K9me2 DNA
BF
H3K9me3
DNA
H3K9me3 DNA
BF
H3K27me2
DNA
H3K27me2 DNA
BF
H3K27me3
DNA
H3K27me3 DNA
BF

Fig S3:

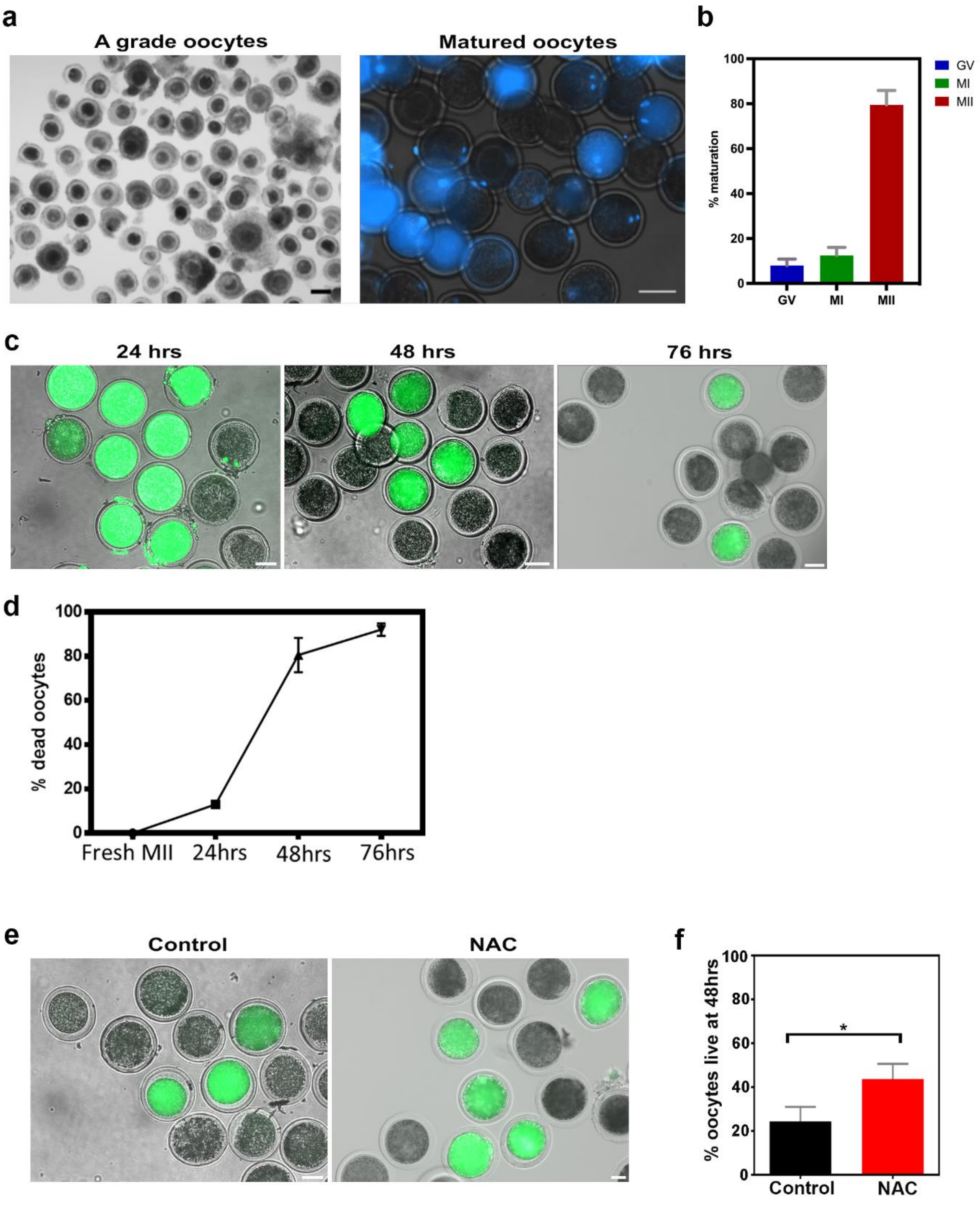
a
A grade oocytes
Matured oocytes
b
% maturation
100
80
60
40
20
0
GV
MI
MII
c
24 hrs
48 hrs
76 hrs
d
% dead oocytes
100
80
60
40
20
0
Fresh MII
24hrs
48hrs
76hrs
e
Control
NAC
f
% oocytes live at 48hrs
100
80
60
40
20
0
*
Control
NAC

Fig S4:

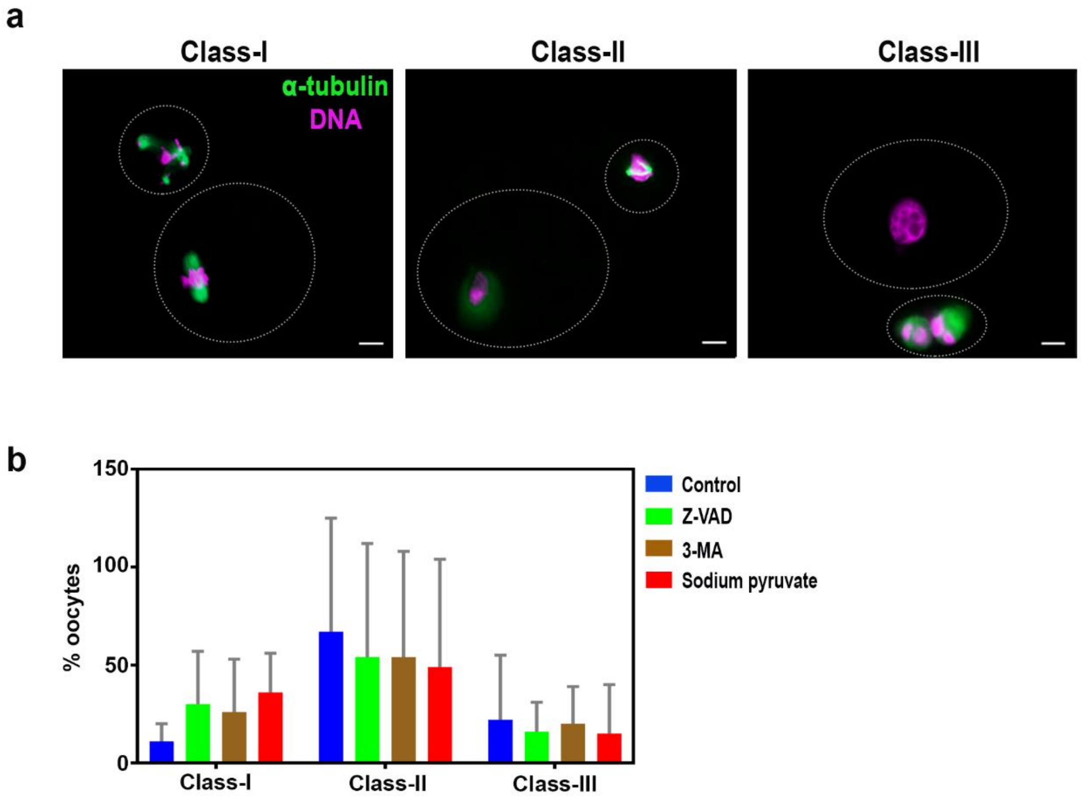
a
Class-I
Class-II
Class-III
α-tubulin
DNA
b
% oocytes
150
100
50
0
Class-I
Class-II
Class-III
Control
Z-VAD
3-MA
Sodium pyruvate
